# Putting the substance in substantial evidence: an evidence-based approach to flexible drug regulation

**DOI:** 10.3389/fmed.2025.1337890

**Published:** 2025-03-20

**Authors:** Emanuel Krebs, Tania Bubela, Melanie McPhail, Christopher McCabe, Dean A. Regier

**Affiliations:** ^1^BC Cancer, Vancouver, BC, Canada; ^2^Faculty of Health Sciences, Simon Fraser University, Burnaby, BC, Canada; ^3^Centre for Public Health and Queen’s Management School, Queen’s University Belfast, Belfast, Ireland; ^4^University of British Columbia, Vancouver, BC, Canada

**Keywords:** substantial evidence, lifecycle regulation, drug regulation, value of information, evidentiary uncertainty

## Abstract

For new drugs or indications, substantial evidence of clinical effectiveness is required for market authorization. In most jurisdictions, substantial evidence is not explicitly defined. Health regulators exercise discretion and are increasingly tolerant of earlier or less mature evidence. To align with flexible evidentiary standards, we argue for the adoption of a principle and, context-based approach to the evidence threshold. Our approach aims to balance the potential benefits and harms of accelerated authorization, low therapeutic value, and safety, based on a value of information (VoI) framework. In our VoI framework, substantial evidence exists when the expected net health value of further research is less than or equal to zero. We operationalize this approach through two case examples that mirror real-time decision factors such as uncertainty, risk preferences and time inputs. As the evidentiary assessment landscape shifts towards flexibility, iterative and clearly defined approaches to risk assessment are warranted. Clarity will stimulate transparency and accountability for both stakeholders and regulators.

## 1 Introduction

To gain health market authorization, most health regulators require drug manufacturers to submit substantial evidence of clinical effectiveness, in addition to meeting other safety, quality, and procedural requirements. Substantial evidence is not defined in legislation or regulations in Canada or the United States, but it has been interpreted as requiring two, well-controlled trials (1). Despite this, both Health Canada and the United States Food and Drug Administration (FDA) maintain discretion to accept alternative evidence thresholds in certain circumstances. For example, FDA has discretion to approve a drug based on one clinical trial if deemed sufficient and if supported by confirmatory evidence, or if a single trial meets certain characteristics, including “a statistically very persuasive finding” (2). Additionally, through the Accelerated Approval pathway, FDA can approve new drugs based on clinical trials using surrogate endpoints (3). In Canada, under the Notice of Compliance with Conditions (NOC/c) policy, Health Canada requires only promising evidence rather than substantial evidence for drugs that address an unmet need or provide an improvement in the benefit–risk profile over existing therapies. Promising evidence may include the use of non-validated surrogate markers or Phase 2 studies not supported by a randomised study design (4).

Beyond these alternative pathways, health regulators are increasingly tolerant of earlier or less mature clinical evidence when assessing regulatory submissions (5). Concurrently, the direction of regulatory reforms and clinical norms indicate a drift from a threshold based on 2 or more randomised controlled trials (RCTs). For example, Health Canada recently proposed regulatory amendments authorizing the imposition of terms and conditions on any drug and medical device approval. This reform aligns with a lifecycle regulatory approach that relies on evidence generated and submitted in the post-market phase (6). Though the Notice of Intent states that imposing terms and conditions should not enable the submission of suboptimal or deficient drug submissions, it is unclear whether Health Canada will permit deviation from its current substantial evidence threshold. Further, health regulators are publishing guidance and recommendations for the submission of real-world evidence to support regulatory authorizations (7).

Based on these trends towards greater flexibility in assessing evidence of clinical effectiveness for pre- and post-market studies and interest in non-traditional evidence, we argue for the adoption of a principle-based definition of the evidence threshold. Our proposed framework can ameliorate some of the concerns associated with evolving regulatory paradigms (8–10) by providing structure for flexible regulatory evidence assessments.

## 2 Redefining substantial evidence

Changing regulatory paradigms present an opportunity to rework the definition of substantial evidence. RCTs are considered the gold standard in determining causal changes in safety and efficacy. RCTs may not be feasible in some contexts, including in rare diseases. RCTs may not result in high quality or meaningful evidence due to power (e.g., limited sample size) and design (e.g., inappropriate comparator) (11). In response, we suggest a contextual approach to evidence assessment at the time of authorization. Our approach recognizes the legitimate concerns of decision-makers in evaluating the trade-off between health gains foregone by current patients due to delayed regulatory approval and the health loss to future patients associated with the risk of making the wrong decision. The wrong decision results from granting market access to a technology that is subsequently shown not to deliver the promised health gains or that harms patients. Our approach balances the potential benefit of accelerated patient access to new drugs against the potential harm inherent in evidentiary uncertainty. Such harm is real. Research has established that most of the drugs authorised by Health Canada and FDA under conditional or accelerated approval pathways were subsequently rated as having low therapeutic value and are also more likely to have serious post market safety problems (12–15).

**Figure 1:**
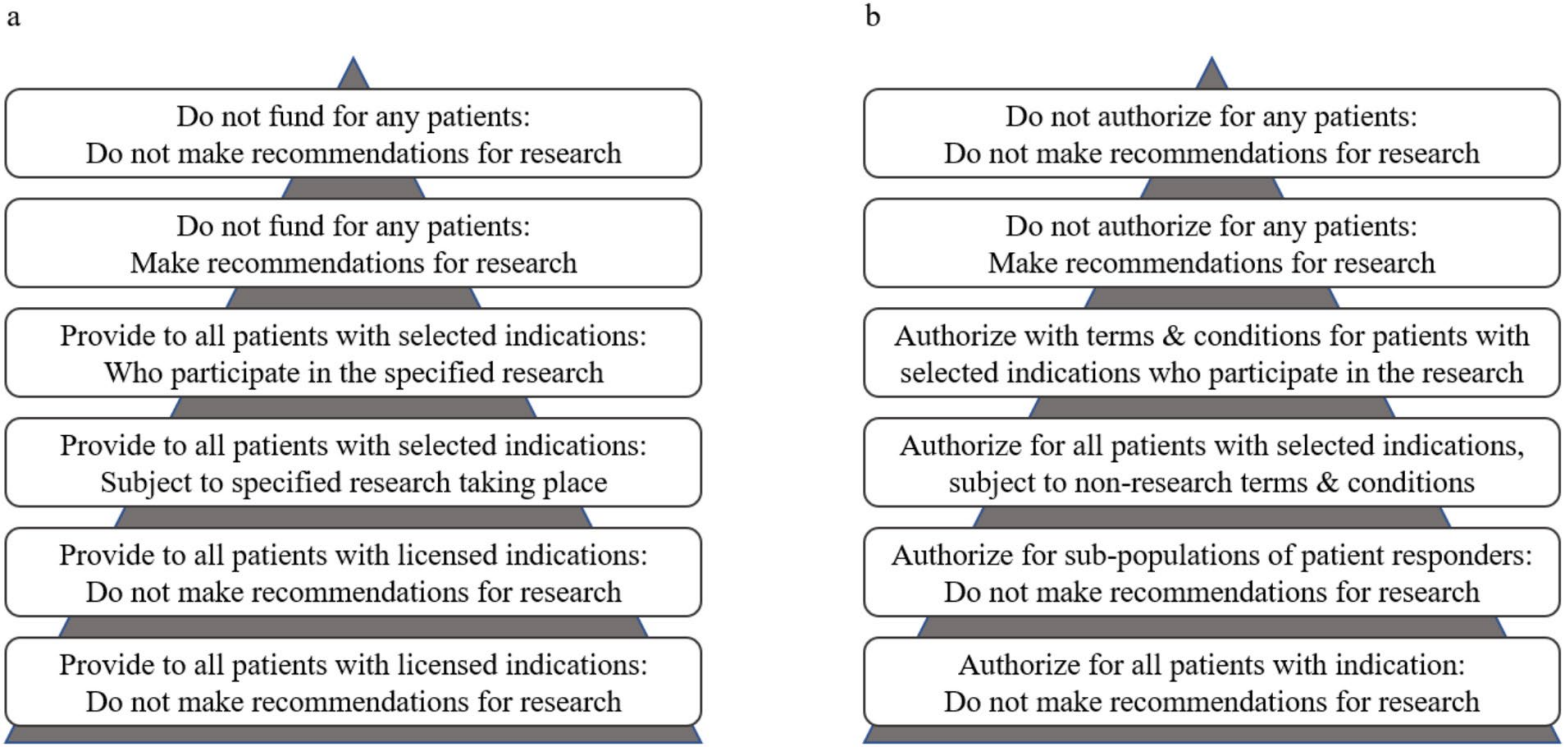
Reimbursement decision options **(a)** based on Edlin et al. (19) for coverage with and without evidence development applied to regulatory decision options **(b)** with and without terms and conditions that enable early access to specified patient populations. The triangles represent the size of the patient populations relative to the reimbursement or access decision.

Decision science explicitly characterises trade-offs. These approaches to the incorporation of uncertainty about trade-offs, specifically the value of information framework (VoI) (16), inform our definition of substantial evidence. The utility of VoI analyses is well established in the context of reimbursement decision processes (17). This approach aligns with the risk-based regulatory paradigm that Health Canada and FDA are pursuing, because it explicitly balances the risks and benefits of immediate versus delayed market access. Our VoI-informed definition of substantial evidence would enable regulators to consider the relative value of (1) allowing access to patients to a new technology without conditions, (2) allowing access to a new technology within the confines of post-market evidence collection (i.e., a conditional approval), (3) delaying access to allow further research within the confines of a later-stage clinical trial, (4) denying market authorization (18). These regulatory options mirror those for reimbursement, which range from full access, to access with evidence development, to delayed reimbursement to allow for further research, to not funding (Figure 1).

Based on the VoI framework, we propose the following definition of substantial evidence:

*Substantial evidence is deemed to exist where the expected loss of health to the indicated patient group due to delayed access to the new technology required for additional research is greater than the gain (or loss avoided) in health that is expected to accrue to the same patient group if additional research were available now. In other words, for substantial evidence to exist, the expected net health value of further research should be less than or equal to zero*.

This definition incorporates a number of health-related factors:

- The number of people who will be affected by the decision to delay access to require more research
- The number of people who would benefit from a decision made with more certain evidence
- The magnitude of expected benefit given existing evidence
- The expected loss of health for people who are denied access to the therapy until additional research reports,
- The expected magnitude of harm from making the wrong decision, given existing evidence

## 3 Case examples of the VoI framework for defining substantial evidence

We provide two hypothetical examples for operationalizing our definition of substantial evidence. The first case example illustrates a risk neutral decision maker, and the second case example assumes risk seeking preferences. In applying our definition, we start by describing the context of the regulatory decision. Sponsors must identify the health need addressed when seeking market authorization for the use of their technology on market. The need may be in terms of prevention, diagnosis, screening, monitoring, morbidity and/or or mortality. Ideally, sponsors provide the regulator with evidence of the health burden the technology aims to address and evidence to support the claim that the technology addresses the health need. By its nature, this evidence will be uncertain. While the expectation based upon the evidence is that the new technology will reduce the health burden, the same evidence allows the regulator to characterise the probability that the new technology will not achieve that benefit.

To illustrate the nature of the decision problem facing the regulator, in both our case examples we use a cure as the endpoint. In reality, the efficacy of the new technology is determined using appropriate, pre-determined endpoints, defined in consultation with the patient community, and may fall short of a cure. Our definition applies equally to different outcomes, for example the number of patients without disease progression. The paradigm shift in cancer treatment represented by precision oncology, which uses ‘omics data to inform treatment independent of cancer type, serves as an example of the real-world applicability of our framework. Regulatory decisions in precision oncology are often made on less mature clinical evidence and our framework can help regulators make informed, evidence-based decisions about trade-offs in regulatory options. We also assume a standard of care treatment is already available on market in our examples, for ease of interpretation, but this analysis could be applied in the absence of an established alternative therapy or when there are no existing treatment options.

**Figure 2:**
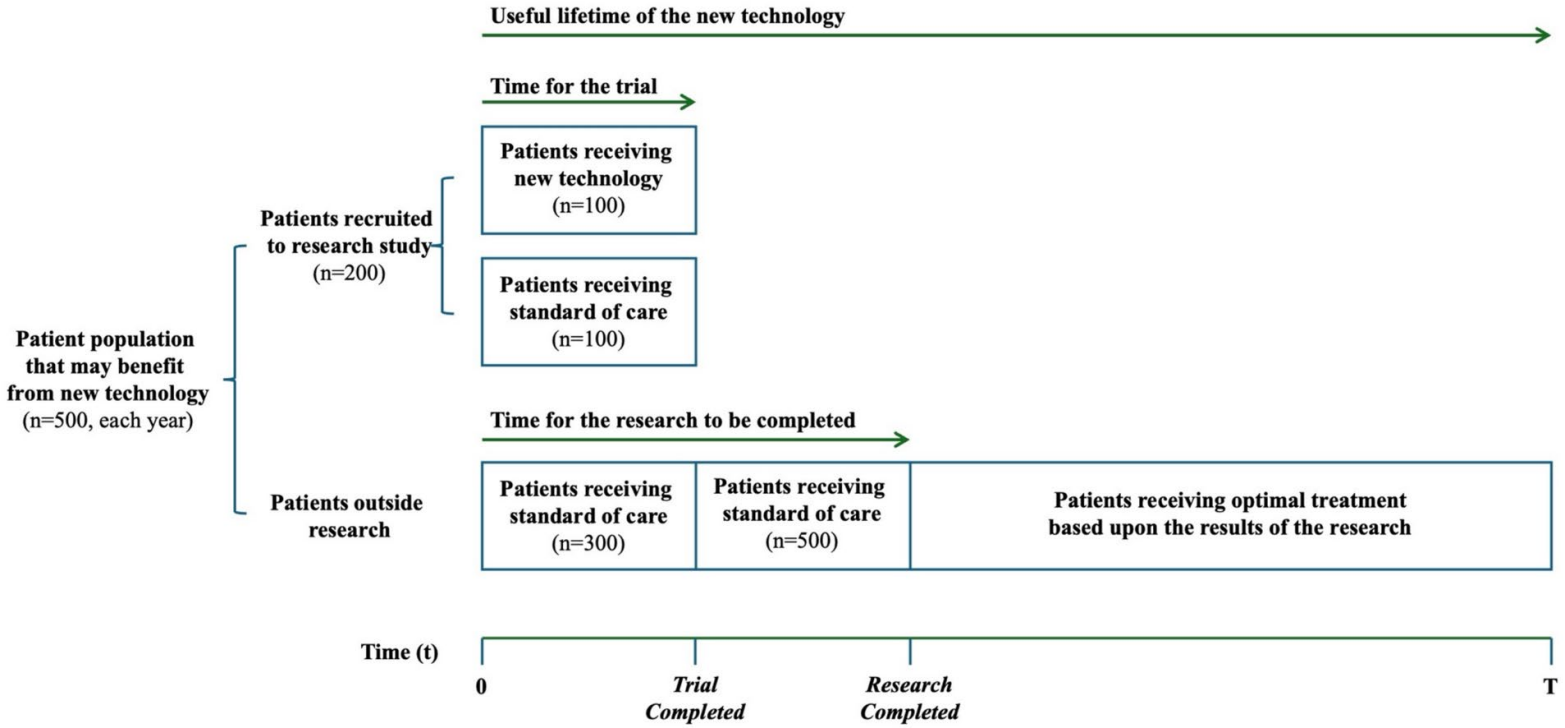
Patient population and how further research is conducted in our case examples. Representation of the value of information framework, based on Edlin et al. (19).

The patient population and how further research is conducted is the same in both examples (Figure 2). The patient population that may benefit from the new technology (NT) is equivalent to 500 patients each year. We assume that 200 patients will be recruited in the trial, and the 300 other patients will receive standard care outside of the trial. In the trial, 100 patients are randomized to receive NT and the other 100 receive standard of care (SoC). We assume the trial will last 1 year, and that the time for the research to be completed and reported will be 2 years. In the second year, after the trial has completed but results have not yet been reported, all 500 patients will receive SoC outside of the trial. Once the trial has reported, all patients receive the optimal treatment based on the results of the research. Our imagined health gain from a successful cure is 10 years of good quality life, irrespective of which treatment achieves the cure. The expected health gain from each treatment is calculated as 10 years, multiplied by the probability that it achieves a cure. We report health gains in present value using a 3% annual discount rate. Lastly, we assume a useful lifetime for NT of 10 years.

### 3.1 First case example: risk neutral decision maker

**Figure 3:**
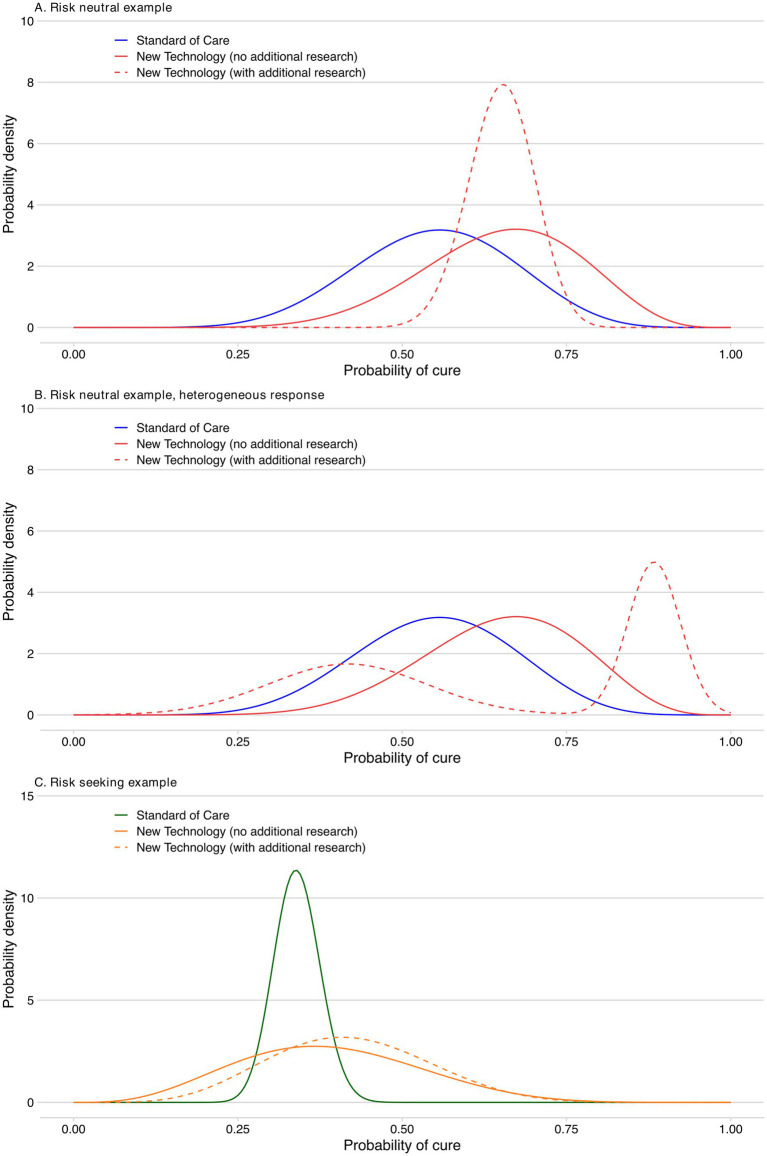
Probability of a cure for the new technology compared to standard of care. **(A)** Risk neutral example. **(B)** Risk neutral example, heterogeneous response. **(C)** Risk seeking example.

Imagine that a trial comparing a NT and SoC reports the probability of NT curing the target clinical indication is 0.65, with a standard deviation of 0.12 (Figure 3A). SoC has a probability of cure of 0.55, with a standard deviation of 0.12. Assuming a beta distribution, we can use these means and standard deviations to illustrate the probability distributions for the effectiveness of the two treatments. If we use the expected value (i.e., mean) to guide our decision between the NT and SoC, we should choose the NT. The probability of being cured is higher with the NT than SoC. However, there is significant uncertainty about which treatment is more likely to produce a cure. For example, there is a 1% chance that the probability of cure is lower than 0.275 with the NT. Conversely, there is a 1% chance the probability of cure for the NT is above 0.81. If we chose the NT, but in reality SoC turned out to have the higher probability of cure, we would have made the wrong decision.

We can use the uncertainty about the probability of cure to consider the risk of making the wrong decision. In Table 1 we have randomly sampled 10 values for the probability of cure from the beta distributions for the NT and SoC. Each of these samples represents a possible State of the World. The correct decision in each state of the world is the one which produces the greatest Health Gain. When we do not consider uncertainty, the decision is made using expected values, and the NT is chosen because the expected health gain is 6.5 years per person treated compared with 5.5 years for SoC. We can examine making the decision based only on the expected values by quantifying the health loss for each State of the World where SoC turns out to produce a greater health gain than the NT. This is the case in States of the World 1, 4, 7, 8 and 9. Across all 10 States of the World, the mean expected health loss due to the uncertainty is 0.22 years.

**Table 1:** Health loss due to uncertainty in the first case example with a risk neutral decision maker, before and after additional research has reported.

	Probability of cure			Health gain (years)^a^			Health loss (years)	
States of the world	Standard of care	New technology, t0	New technology, t1	Standard of care	New technology, t0	New technology, t1	New technology, t0	New technology, t1
1	**0.661**	0.655	**0.755**	**5.80**	5.75	**6.63**	0.05	0.00
2	0.480	**0.681**	**0.600**	4.22	**5.98**	**5.27**	0.00	0.00
3	0.465	**0.713**	**0.640**	4.09	**6.26**	**5.62**	0.00	0.00
4	**0.575**	0.571	0.571	**5.05**	5.02	5.02	0.03	0.03
5	0.526	**0.576**	**0.540**	4.62	**5.06**	**4.74**	0.00	0.00
6	0.505	**0.625**	**0.621**	4.44	**5.49**	**5.46**	0.00	0.00
7	**0.521**	0.510	0.510	**4.58**	4.48	4.48	0.10	0.10
8	**0.740**	0.615	0.709	**6.50**	5.40	6.23	1.10	0.27
9	**0.715**	0.641	0.641	**6.28**	5.63	5.63	0.65	0.65
10	0.312	**0.913**	**0.913**	2.74	**8.02**	**8.02**	0.00	0.00
Average	0.55	0.65	0.65				0.192	0.105
Expected Value	0.55	0.65	0.65	4.83	5.71	5.71		

a Health gains are reported using a 3% annual discount rate.

t0, time of conditional market authorization; t1, additional research reported.

Bold indicates the correct choice; grey cells indicate instances with a health loss given the choice of implementing the New Technology based on expected value of health; green cells indicate instances when further research changed correct choice between t0 and t1, reducing health loss.

Regulators considering a promising but highly uncertain therapy have to decide whether to authorise the technology with the currently available evidence, whether they should delay patient access to allow additional research to be completed, or whether they should authorise the technology on the condition that additional post-authorization research or enhanced post-market surveillance is conducted.^1^ The additional research or post-market surveillance would be expected to reduce the uncertainty and hence the risk of making the wrong decision. In the example above, the research is assumed to reduce the expected health loss due to uncertainty. It is possible to identify the health loss when patient access is delayed to allow the research to take place.

By using such an analysis, decision makers could determine whether the benefit of the research was sufficient to justify its cost, where the cost is not financial but instead the health loss attributable to the delay. If a regulator decides to authorise a therapy conditional on conducting additional research or enhanced post-market surveillance, the type of research arrangement will influence the calculation. For example, if a therapy is authorised conditional on additional research while widely accessible to patients, the cost of research now includes the cost of providing treatment to all patients not in the control arm. Additionally, because recruitment to clinical trials for therapies widely available is challenging, the time it will take to complete the research will be impacted.

We now use the same example to understand how the health loss from the research can be quantified. The expected health loss from delaying patient access to implement the research can be calculated as the difference in the expected health gain between the NT and SoC for the patients who will receive SoC rather than the NT during the 2 years that research is on-going. As illustrated in Figure 2, this includes the 400 patients receiving SoC during year 1, including their expected health loss during the second year, and the 500 patients receiving SoC during year 2. That is a loss of 127.4 years of good health total, in present value (calculated as 1/10 years are lost by 400 patients each year over 2 years plus 1/10 years that are lost by 500 patients during year 2, reported using a 3% annual discount rate).

The value of the research is driven by its impact upon the uncertainty in the evidence base for both the NT and SOC. For the sake of this illustration, we assume that with the new evidence, the standard deviation for the probability of cure from the NT is reduced to 0.05, and the expected value remains the same (Figure 3A).

In Table 1, we update the 10 States of the World for the NT to reflect the updated evidence base and illustrates the impact of the new research expected health loss due to uncertainty. The expected health loss due to uncertainty has decreased by 0.087 years of good health, calculated as the difference between health loss before additional evidence is generated (0.192) and health loss after our analysis shows that new evidence resolves (some) uncertainty (0.105). The 2 years that the research takes to report means that the benefit of the reduced uncertainty will be available for 10 years, and that a total of 5,000 patients will benefit from the more certain decision. The total value of the reduction in the expected health loss due to uncertainty is 0.087 multiplied by 5,000, which equals 435.5 additional years in good health (or fewer years of good health lost).

In this illustration, the expected net health value of the research over the lifetime of the decision is 308.1 years of good health (435.5 fewer years of good health lost—127.4 years of health loss due to delay for conducting further research), assuming future health is not discounted and, which is calculated by subtracting health loss from health benefit (19). In this illustration, the definition of substantial evidence would not be met, because the expected impact on health is greater from delaying access to NT while further research is undertaken. The value of delaying access to allow the research to be undertaken reduces with the size of the clinically indicated population, the risk of making the wrong decision, and the duration of the useful life of the technology.

### 3.2 Heterogeneous response to NT

We also provide an illustration of how our framework for defining substantial evidence can capture heterogeneous response to NT (Figure 3B). For this illustration in the case of a risk neutral decision maker, the patients are characterised by two equal-sized groups with distinct high (0.88) and low (0.42) probabilities of NT curing the target trial clinical indication. This heterogeneous response could represent instances such as variation in treatment adherence or other (unknown) characteristics influencing how patients respond. For simplicity, we have defined the distinct patient subgroups such that the same States of the World and results as presented above (and in Table 1) for a homogeneous response are applicable (i.e., the overall probability of cure remains 0.65). Our framework, without any loss of generality, could of course be adapted to different characterizations of patient heterogeneity. Lastly, the flexibility provided by our framework thus also allows for explicit consideration of the impact of potential placebo and nocebo effects in studies with subjective endpoints.

### 3.3 Second case example: risk seeking decision maker

Regulatory and reimbursement policies over the last few decades suggest that regulators are risk seeking when making decisions. Initially these decisions were most evident in the consideration of treatments for rare diseases. However, recently regulators have been showing risk seeking attitudes in the context of highly prevalent diseases. Recent examples include FDA approvals of novel treatments for Alzheimer’s disease. It is rational for decision makers to adopt risk seeking approaches when conventional therapies have failed to meet the needs of patients. The level of acceptable risk should be informed in consultation with the patient community that may benefit from the novel treatments being considered. To encourage investment in translating the most recent science into novel therapeutics, regulators and payers need to signal that such work will be rewarded by selectively adopting a risk seeking attitude when assessing the value of more research.

To illustrate this, we have applied the VoI approach to a new example in which the SoC has a lower probability of cure (0.34). There is a highly uncertain new therapy that is expected to have a probability of cure of 0.39, and there is a non-trivial possibility that NT is worse than SoC (Figure 3C). A risk seeking attitude by the regulator would mean that the health gain foregone to allow further research would be valued more highly than the expected health loss avoided as a result of undertaking the research.

For ease of illustration, we have set the value of cure for a risk seeking decision maker for a one-year health increase as a function of the uncertainty around the expected value of cure. Specifically, our illustrative value function is V_i_ = E[X_i_]*(1 + *x*_imax_ − *x*_imin_), where V_i_ is the value of intervention *i*, E[X_i_] is the risk neutral expected value of a cure for intervention *i*, *x*_imax_ is the highest random variable in the probability distribution of a cure for intervention i, and *x*_imin_ is the lowest random variable in the probability distribution of a cure for intervention *i*. The term *x*_imax_-*x*_imin_ is the range of the probability distribution. In our example, we calculate that the risk seeking value of a 1 year produced by the NT is 1.48, while the value of a year produced by SoC is 1.11. The more uncertain (riskier) the technology the greater the value that is attached to the health it produces.

In this case example, the value of the health (years of life) foregone while research is undertaken is higher than the reduction in the expected health loss attributable to that research. The total value of the reduction in the expected health loss due to uncertainty is 0.016 (0.091–0.075) multiplied by 5000, which equals 80.0 additional years in good health (or fewer years of good health lost). In this illustration, the expected net health value of the research over the lifetime of the decision is 174.5 years of good health (80.0 fewer years of good health lost —254.5 years of health loss due to delay for conducting further research). When a risk seeking value position is incorporated into the analysis, delaying access to the new therapy to allow further research is not the preferred choice (see Table 2).

**Table 2:** Health loss due to uncertainty in the second case example with a risk seeking decision maker, before and after additional research has reported^a^.

	Probability of cure			Health gain (years)^b^			Health loss (years)	
States of the world	Standard of care	New technology, t0	New technology, t1	Standard of care	New technology, t0	New technology, t1	New technology, t0	New technology, t1
1	**0.300**	0.280	**0.310**	2.93	**3.64**	**4.03**	0.00	0.00
2	**0.360**	0.350	**0.385**	3.51	**4.55**	**5.01**	0.00	0.00
3	0.340	**0.420**	**0.395**	3.32	**5.46**	**5.14**	0.00	0.00
4	**0.280**	0.200	0.210	**2.73**	2.60	**2.73**	0.13	0.00
5	**0.290**	0.270	**0.410**	2.83	**3.51**	**5.33**	0.00	0.00
6	0.380	**0.550**	**0.580**	3.71	**7.15**	**7.54**	0.00	0.00
7	0.390	**0.650**	**0.690**	3.80	**8.45**	**8.97**	0.00	0.00
8	**0.360**	0.340	0.345	3.51	**4.42**	**4.49**	0.00	0.00
9	**0.320**	0.180	0.235	**3.12**	2.34	**3.06**	0.78	0.75
10	0.380	**0.660**	**0.640**	3.71	**8.58**	**8.32**	0.00	0.00
Average	0.34	0.39	0.42				0.091	0.075
Expected Value	0.34	0.39	0.42	3.32	5.07	5.46		

a The risk seeking value of a one-year health increase for the Standard of Care is 1.11 and for the New Technology 1.48.

b Health gains are reported using a 3% annual discount rate.

t0, time of conditional market authorization; t1, additional research reported.

Bold indicates the correct choice; grey cells indicate instances with a health loss given the choice of implementing the New Technology based on expected value of health; green cells indicate instances when further research changed correct choice between t0 and t1, reducing health loss; blue cells indicate instances when the risk seeking assumption changed correct choice from a risk neutral assumption.

For comparison, the value of the research from a risk neutral perspective, i.e., each year of health is valued at 1 irrespective of whether it is produced by the NT or SoC, is 0.105 (0.255–0.150) multiplied by 5,000, which equals 526.8 additional years in good health. The health loss due to delaying access to the NT to allow the research to proceed is 63.7, and the expected net health value of the research over the lifetime of the decision is 463.1 years of good health (526.8 fewer years of good health lost—63.7 years of health loss due to delay for conducting further research). Delaying for research would be the preferred choice from the risk neutral perspective (see Table 3).

**Table 3:** Health loss due to uncertainty in the second case example but with a risk neutral decision maker, before and after additional research has reported.

	Probability of cure			Health gain (years)^a^			Health loss (years)	
States of the world	Standard of care	New technology, t0	New technology, t1	Standard of care	New technology, t0	New technology, t1	New technology, t0	New technology, t1
1	**0.300**	0.280	**0.310**	**2.64**	2.46	**2.72**	0.18	0.00
2	**0.360**	0.350	**0.385**	**3.16**	3.08	**3.38**	0.08	0.00
3	0.340	**0.420**	**0.395**	2.99	**3.69**	**3.47**	0.00	0.00
4	**0.280**	0.200	0.210	**2.46**	1.76	1.84	0.70	0.62
5	**0.290**	0.270	**0.410**	**2.55**	2.37	**3.60**	0.18	0.00
6	0.380	**0.550**	**0.580**	3.34	**4.83**	**5.10**	0.00	0.00
7	0.390	**0.650**	**0.690**	3.43	**5.71**	**6.06**	0.00	0.00
8	**0.360**	0.340	0.345	**3.16**	2.98	3.03	0.18	0.13
9	**0.320**	0.180	0.235	**2.81**	1.58	2.06	1.23	0.75
10	0.380	**0.660**	**0.640**	3.34	**5.80**	**5.62**	0.00	0.00
Average	0.34	0.39	0.42				0.255	0.150
Expected Value	0.34	0.39	0.42	2.99	3.43	3.69		

a Health gains are reported using a 3% annual discount rate.

t0, time of conditional market authorization; t1, additional research reported.

Bold indicates the correct choice; grey cells indicate instances with a health loss given the choice of implementing the New Technology based on expected value of health; green cells indicate instances when further research changed correct choice between t0 and t1, reducing health loss.

## 4 Regulatory implications

A flexible approach to determining the threshold of substantial evidence for clinical efficacy has legal and regulatory implications, requiring concomitant safeguards to ensure definitional and process transparency, accountability, and consistency in decision-making. Adopting a more dynamic definition of substantial evidence will affect regulatory decision-makers, forcing them to make regulatory decisions in the context of greater evidentiary heterogeneity. Transparency in the assessment and use of evidence in regulatory decision-making over time mitigates the uncertainty for patients, physicians, payors, and other stakeholders associated with greater variation in evidence used to support regulatory decisions. An evidence-based, dynamic definition of substantial evidence will also impact reimbursement decision-makers, physicians, and patients, allowing them to make decisions in a context of greater transparency, mitigating concerns with flexible and discretionary regulatory approaches. To facilitate knowledge diffusion, prompt and proactive disclosure of pre-market evidence assessments should be built into the regulatory framework. This will require updated infrastructure and novel communication mechanisms.

Iterative regulatory information and clinical evidence poses challenges for labeling. How informational changes will be made to avoid confusion and uncertainty will need to be clarified in the proposed regulatory framework. Additionally, off-label prescribing under a more flexible regulatory paradigm may become problematic. Restricting off-label use has been proposed as a potential mitigation mechanism, however, legal and pragmatic barriers to restricting off-label use suggest that a more moderate approach may be more appropriate, such as routine documentation of patients treated off label to enable enhanced monitoring (20, 21).

The proposed reforms also provide an opportunity to consider the explicit and expanded incorporation of qualitative and quantitative patient preferences in the regulatory deliberation process (22, 23). Introduction of additional regulatory flexibility should consider patient preferences for the risk–benefit trade-offs in light of outcomes uncertainty, which may vary depending on the context and specific patient subgroups (24, 25).

Documented challenges experienced to date with life-cycle regulatory approaches need to be addressed to ensure the sustainability and success of this approach. For example, while life-cycle regulatory decisions rely on the ability to conduct post-market trials, such trials are often delayed or not completed at all (26, 27). Several mechanisms have been proposed to promote compliance with post-market trials, including stronger fines and penalties, public timelines, mandatory publication of final results, requiring confirmatory trials to be underway at the time of authorization, and automatic expiration of the authorization (28, 29). Additionally, it is imperative that post-market trials are appropriately designed with clearly defined outcomes to address evidentiary uncertainties (30, 31).

The modernization of pre-market evidence assessment processes can support the use of more a flexible definition of substantial evidence while maintaining accountability and protection of patient health. Over the last decade, global health regulators have expressed interest in harmonizing benefit–risk assessments to minimise regulatory duplication and increase transparency. In 2008, Health Canada, in collaboration with the Australian Therapeutic Goods Administration, Swissmedic, Singapore’s Health Sciences Authority and the Centre for Innovation in Regulatory Science, formed the Consortium on Benefit–Risk Assessment (COBRA), and developed a methodology for conducting benefit–risk assessments, in alignment with the 2012 Universal Methodology for Benefit–Risk Assessment (UMBRA). The UMBRA framework consists of 8 standardized steps for conducting benefit–risk assessments, accompanied by a proforma (32, 33). Proactive disclosure of uncertainties and risk–benefit assessments at the time of approval and ongoing disclosure of post-market evidence generation is critical for encouraging transparency and consistency in decision-making and improving communication to stakeholders (34).

Despite Health Canada’s involvement in COBRA, it remains the only regulatory authority studied that does not use a documented benefit–risk assessment in drug submission assessments (35). Notable omissions include identification of outstanding issues, regulatory history, overall summary of risks, weighting and valuing benefits and risks (31). Possibly, however, Health Canada uses an unpublished standardized benefit–risk assessment during the pre-market evaluation. Similarly, FDA has failed to adopt a comprehensive benefit–risk assessment protocol, despite the 2012 recommendation from the Institute of Medicine to adopt a Benefit and Risk Assessment and Management Plan, and to implement a lifecycle regulatory approach to evidence assessment while promoting transparency in decision making. Conversely, FDA initiatives aimed at promoting an increased use of real-world evidence, including recent draft guidance promoting the integration of trial-related activities into routine clinical practice, suggest promising avenues for how our framework could be effectively implemented to support regulatory decisions (36, 37). The adoption of the proposed quantitative approach to characterising evidentiary uncertainty, and how that is incorporated into regulatory decision making would be a positive development in the transparency and consistency of regulatory decision making.

## 5 Conclusion

We strongly support the adoption of more iterative approaches to risk assessment. However, lessons to date from the application of flexible regulatory pathways suggest that their inconsistent application results from a lack of clarity in the scope and substance of evidence assessments. Accordingly, we recommend the development of clear definitions and a more comprehensive, contextual assessment framework to justify whether regulatory submissions have met the market authorization threshold of substantial evidence. Clarity will enhance transparency and provide greater certainty for stakeholders, including patients and manufacturers. Adopting a more structured benefit–risk evaluation process and communicating its findings will enhance accountability under the more flexible evidentiary assessment approach pursued by drug and device regulators.

## Data Availability

The original contributions presented in the study are included in the article/supplementary material, further inquiries can be directed to the corresponding author.

## Footnotes

^1^Allowing full market access whilst the evidence is created increases the cost of making the wrong decision, and therefore, all things being equal is less efficient in proportion to the excess of the clinically indicated population over the sample size required for the population.
